# Self-care strategies for emotional distress among young adults in Catalonia: a qualitative study

**DOI:** 10.1186/s13033-015-0001-2

**Published:** 2015-03-10

**Authors:** Maria-Antonia Martorell-Poveda, Angel Martinez-Hernáez, Natalia Carceller-Maicas, Martin Correa-Urquiza

**Affiliations:** Department of Nursing, Universitat Rovira I Vigili, Avda. Catatalunya, 35, 43002 Tarragona, Spain; Department of Anthropology, Philosophy and Social Work, Universitat Rovira I Vigili, Avda. Catatalunya, 35, 43002 Tarragona, Spain

**Keywords:** Adolescents, Mental health and illness, Self-care, Self-help, Social support, Young adults

## Abstract

**Background:**

Emotional distress is common in adolescence, and self-care strategies are frequently preferred to address it. The aim of this article is to analyze the self-care strategies among adolescents and young people diagnosed with depression or with self-perceived depressive distress in Catalonia using a qualitative design.

**Methods:**

We analyzed the self-care strategies of 105 young people (17–21 years of age) in Catalonia who had participated in a national survey on adolescents. The sample was divided into thirds, with 37 who had a previous diagnosis of depression, 33 who had self-perceived emotional distress, and 35 controls. The participants’ narratives on self-care strategies for emotional distress were elicited through in-depth semi-structured interviews. The data were managed using ATLAS-Ti 6.5 software18. We applied hermeneutic theory and the ethnographic method to analyze the interviews.

**Results:**

The ten self-care strategies identified in the analysis were grouped into four areas covering the various pathways the young people followed according to whether they had a diagnosis of depression or their depressive distress was self-perceived. The young people feel responsible for their emotional distress and consider that they are capable of resolving it through their own resources. Their strategies ranged from their individuality to sociability expressed through their relationships with others, membership of groups or other self-care strategies (relaxation, meditation, naturopathy, etc.).

**Conclusions:**

The study results highlight the importance of sensitivity in considering young people’s self-care strategies as another option in the care of emotional distress.

**Electronic supplementary material:**

The online version of this article (doi:10.1186/s13033-015-0001-2) contains supplementary material, which is available to authorized users.

## Background

Emotional distress is common in adolescence, and lay strategies are frequently chosen to cope with it. Adolescence is a critical phase in the construction of personal and social identity, and vulnerabilities associated with it include depression, anxiety and negative mood states [1-5]. Similarly, research has shown that adolescents tend to reject and have negative attitudes toward seeking professional help, preferring self-help to treat these problems [2,6-9]. However, there is evidence that knowledge is lacking about which self-help strategies are effective for mental health problems, especially in cases where symptoms are less severe [10].

A recent systematic review [11] points to the need to identify the actions adolescents can use in their everyday lives to reduce their risk of depression without professional help or other formal intervention. The results of the review suggest that there is a relationship between substance use (alcohol, tobacco, cannabis, other illicit drugs and polydrug use), healthy diet and weight, coping strategies, sleep (sufficient hours and quality), and depression, and that these factors can be both risk factors for or protectors against symptoms of depression. Other actions identified as possible protective factors for depression among adolescents are dating during adolescence, media use, physical activity and sport, relationships with positive peers, and self-disclosure to parents.

Previous studies have also shown that adolescents and young adults mainly turn to informal networks such as friends and family [12-14] or, although to a lesser extent, choose lifestyle-related strategies or complementary therapies [15].

In all complex societies we can identify lay care strategies that lie outside the boundaries of professional services and care. Authors have used a range of terms to refer to lay care, such as self-help [16], self-care [17], social support [18], or informal care [19]. The conceptual diversity in the literature suggests that this type of care covers a wide range of resources and practices. In fact, a recent literature review [20] identifies the following wide range of strategies used by adults with mental health problems: employment, education, creative activity, physical exercise, healthy living, structured routine, and spirituality. These authors propose a model of self-care in mental health based on the inclusive use of the concept that also encompasses other concepts such as recovery, self-management and self-help. This model, in which the individual is placed at the center, shows reciprocal relationships with self-care support, self-care behaviors and strategies, and well-being and functioning. Leaving aside discussions on the suitability of one term over another, or the extent of their scope, it seems clear that self-care is a common practice with a particular centrality in every society [21,22].

It is noteworthy, however, that in general the research to date has tended to quantify the frequency of informal care and self-help. The undeniable interest of such data nevertheless does not preclude the relevance of exploring the issue from a qualitative perspective. With good reason, the World Health Organization [5] proposal highlighted the importance of understanding self-care from a position of experience and lay knowledge. In the same vein, other authors [20] highlight the importance of considering the experiences of people with mental health problems to understand self-care in mental health. In socio-anthropological terms, this essentially means approaching the contents and meanings of young people’s actions from first-hand personal experiences; and in this context, discovering what lay strategies young people use to cope with their situations of emotional distress.

Our aim in this article, therefore, is to analyze the self-care strategies among adolescents and young people diagnosed with depression or with self-perceived depressive distress in Catalonia. To this end we considered the repertoire of activities and resources young people use to cope with, control, alleviate, endure and resolve their situations of distress without the intervention of professional services [23-25].

## Methodology

This research is the first systematic study in Spain and Catalonia into the processes of avoidance of professional mental health care services and the use of lay resources among adolescents and young people with depression or associated factors, using qualitative methodology.

### Design and sampling

We conducted 105 face-to-face in-depth interviews with young people selected from the *Families and Childhood Panel*, a national survey begun in 2006 with adolescents resident in Catalonia who were born between 1990 and 1993; the survey incorporated a new cohort every year until 2009. Information was collected on negative mood states using a self-administered scale of distress in waves 2 and 3, and on diagnoses of depression or anxiety (reported by parents) in waves 1 and 4. The self-administered scale of distress identified different mood states on a five-point Likert-type scale (ranging from ‘never’ to ‘often’). Chronic states of sadness, nervousness, and loneliness were statistically associated with a diagnosis of depression in all the waves (2 and 3) in which the scale was applied. All waves also included information on consumption of psychoactive substances, sociability, and familial and educational variables.

The subsample was recruited from all over Catalonia, including rural areas, using the propensity matching score technique to yield three groups of 50 participants each: one with depression or anxiety diagnosed by a health professional in the first or fourth wave of the *Families and Childhood Panel*, as reported by the parents in response to a direct question; a second group with self-perceived depressive distress (chronic states of sadness, nervousness, and loneliness) in the second and third wave but without a diagnosis of depression or anxiety; and a control group with neither self-perceived distress nor a psychiatric diagnosis. Sample attrition occurred in cases of change of residence, inability to contact the subject, or subjects who declined to be interviewed, and in the end 105 subjects were interviewed: 37 with a diagnosis (GD), 33 with self-perceived distress (SPD), and 35 controls (GC) (see Table 1). The number of cases was sufficient to satisfy the principle of saturation in qualitative research.

**Table 1:** **Sociodemographic characteristics**

		**Groups**			**Total**
		**Participants with diagnosis**	**Participants with mental distress**	**Control participants**	
		**N**	**N**	**N**	
**Sex**	Male	13	9	11	33
	Female	24	24	24	72
	Total	37	33	35	105
**Age**	17 years	5	3	7	15
	18 years	10	6	5	21
	19 years	12	10	9	31
	20 years	7	9	12	28
	21 years or older	3	5	2	10
**Family income level**	Under 18000€	6	5	8	19
	From 18001 to 36000 €	18	13	13	44
	36001€ or more	9	7	5	21
	Don’t remember	4	7	8	19
**Family structure 2010**	Single-parent family	11	7	14	32
	Two-parent or reconstituted family	17	19	19	55
	No information	9	7	2	18

The participants were young people aged between 17 and 21 years, 68.6% of whom were young women, and 31.4%, young men. All were resident in Catalonia, including rural areas. The sociodemographic characteristics of the missing subjects were not significantly different from those of the subjects interviewed.

Interviewing was carried out between March and October 2011 at the convenience of the participants, who were contacted by telephone.

The study was approved and monitored by the Spanish Ministry of Science and Innovation and the *Fundació La Marató de TV3*, a Catalan not-for-profit foundation that raises funds for biomedical research. The study procedures were approved by the ethics committee of the *Fundació Congrés Català de Salut Mental* (Catalan Mental Health Congress Foundation), an interdisciplinary entity for the promotion of mental health, and carried out in accordance with the ethical standards established by the Helsinki Declaration of 1964. Each participant and one adult with parental responsibility provided written informed consent.

### Data collection

The participants’ narratives on self-care strategies for emotional distress were elicited through in-depth semi-structured interviews. For this interview, we designed an initial vignette (Additional file 1) in which an imaginary 17-year-old, John or Mary, showed signs of depression according to DSM-IV-TR criteria, but without offering possible reasons or causes. The interviewer did not use the word ‘depression’ or any other diagnostic category. The interviews were carried out in Spanish or in Catalan, depending on the subject’s mother tongue. The character in the sketch was always of the same sex as the person interviewed.

After reading the sketch, the interviewer proceeded to the questionnaire, which took approximately an hour and a half to complete. Informants were asked to explain what they thought was happening to John or Mary, and the interview focused progressively, and in a retrospective and reflexive manner, on the subject’s own experience of emotional distress during transitional aged youth.

The 11 interviewers, all of whom were researchers in medical anthropology and/or psychology, participated in two working sessions to unify criteria and coordinate the dynamics of fieldwork and interviews. Finally, each interviewer wrote up a reflexive evaluation of every interview completed.

### Data analysis

Interviews were audio or video recorded and transcribed verbatim. The data were managed using ATLAS-Ti 6.5 software18. Following an initial analysis to identify the principal themes in the data obtained, we began an independent analysis and subsequently discussed our observations to create an initial coding framework in accordance with the principles of hermeneutic theory and the ethnographic method, including discovery of emic or native typologies [26,27], that is, explanations and meanings from the point of view of the young people themselves.

Four researchers, the authors of this article, held face-to-face discussions to produce an initial thematic analysis and identify the main codes in the data. We sought the views of a fifth member of the research team when discrepancies arose in this process. We then reviewed all the interview transcripts, and revised and applied the codes to the data using constant comparison, identification and analysis of exceptions, as well as reflexivity and comparative peer review, until content saturation was achieved. When researchers were unsure how to code data we resolved issues and modified the code through team discussion by e-mail. Some qualitative data were transformed into binary matrices and analyzed with Ucinet 6. 454 software, which yielded the relationship between the self-care strategies used and the three subgroups identified in the study.

## Results

Our analysis of the interviews clearly revealed an attitude among young people to seek help in their social network and to use other self-care resources as an alternative, and to a lesser extent as a complement, to professional treatment. The actions and resources young people use were summarized in ten categories, reported in Table 2, that we grouped for analytical purposes into the four main areas we identified from the associations among the items.

**Table 2:** **Self-care strategies by subgroups**

	**Female diagnosis**	**Female self-perceived distress**	**Female control**	**Male diagnosis**	**Male self-perceived distress**	**Male control**	**Total**
Self-care	11	6	12	8	4	6	47
Friends	20	18	23	11	7	7	86
Others	11	8	6	4	4	3	36
Parents	9	10	11	6	5	4	45
Adults	3	3	3	0	4	1	14
Girlfriend/boyfriend relationship	4	3	3	1	1	1	13
Relatives	8	5	16	4	6	6	45
Sport	3	7	10	1	7	8	36
Artistic	11	4	4	5	4	1	29
Alternatives	2	3	1	2	0	1	9
Total	11	9	12	5	5	4	46

**Figure 1:**
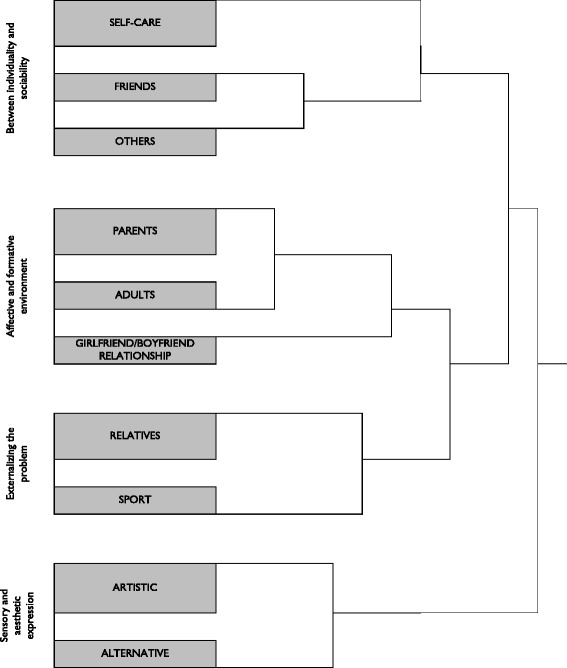
**Cluster diagram.** Note: Johnson’s Hierarchical Cluster. Method: Average between all pairs. Prepared using UCINET 6.454.

These are represented in the form of clusters in Figure 1. These self-care resources can be combined and are common to all three groups of young people in the study. We describe the four areas referred to below:

1. The first area includes the resources that reinforce the young person’s individuality in his or her life cycle stage, in that they aim to maintain personal autonomy in resolving conflicts. At the same time we observe some socialization of problems with friends, as the main motives for emotional distress also originate in this social context and it is where their solution can be found.
2. The second area concerns the adult world, and in particular, the emotional and educational dimension. Adults, essentially parents and teachers, offer a type of support in which their presence and ‘unconditional love’, particularly parental, is meaningful for the young person during the self-care process. In some way, attention from the adult world entails that the young person is accompanied into his or her future. This area also includes the boy/girlfriend relationship in that it offers affection and trust as a support to cope with the situation of adversity.
3. The third area involves taking the problem beyond close social networks, channeling the stress that the situation of emotional distress causes through one’s body in a physical activity or sport, and through trust and conversation with a close relative.
4. Finally, the fourth area highlights self-care strategies that link the young person’s internal and external worlds through sensory and artistic expression.

### Between individuality and sociability

Young people express their concern to maintain personal autonomy and self-control in the process of seeking help for emotional distress. This perspective arises in their narratives when they refer to the need they feel to look for the solution to their problems within themselves and by themselves. For them this represents a strengthening and maintaining of their individuality. In the words of some informants: ‘It’s like you have to be yourself. Help from outside can help you but you have to be more yourself as a person, you have to realize yourself’. (Young man, SPD). ‘My attitude was that I had to help myself: I don’t want anyone else interfering in my life’. (Young woman, GC).

When you have a problem, well, if you say, like, I’m not feeling great or whatever, but afterwards it’s you that lives with it. Other people can help you at a certain moment but when you’re on your own and the way you deal with the problem it’s for yourself and not for anyone else. (Young woman, GD).

Mobilizing personal resources takes on different connotations among the young people. Some literally expressed the need to act and move themselves, believing that no one else can get them out of their situation. For others it is a question of ‘emptying out the problems’, in search of their desired happiness internally and within the self. One of the informants from the self-perceived distress group used the metaphor of silence to explain this process of internalization. According to his testimony, when one experiences silence and breaks away from all background noise, one realizes how it affects one’s situation and becomes aware of what needs to change and what to do to improve it. In his opinion, being silent helps one to be alone with oneself, allowing one to find a solution to the problem. Moreover, this strategy can be used whenever and wherever the person wants.

Another strategy some young people use to establish this process of internal communication is to express their experiences through writing. One young man said, ‘I write things down on paper and after that I react and see what to do or how to act’. (Young man, SPD).

The young people seem to agree substantially with the idea that they can only rely on themselves if things take a turn for the worse. They also stated that they only ask others for help when they have no alternative and cannot resolve the situation using their own resources. Likewise, the narratives also revealed that the strategies for dealing with situations of adversity can go beyond personal resources. The young people shared the idea that as time passes, they cannot stay trapped inside the problem and they need to share the experience. In their opinion, ‘when you talk about things, they get smaller. So, the more people you tell, the sillier it seems’. (Young woman, GC).

When a young person decides ‘to come out of him or herself’, he or she tends to socialize the process in the context of friendships, the same space in which, for the majority, the distress originates. They considered that there is ‘always mutual support in friendships’, (Young man, GD), where talking and encouraging the other is one of the main resources with which to face the situation of adversity. In fact, the young people considered their friends to be one of the main sources of psychological support in everyday and crisis situations alike. The activities they share with friends include having a coffee, going for a walk or to the movies, and with them they can ‘let off steam’, trust and seek understanding when they are experiencing a situation of failure or criticism. It is their friends who cheer them up when they are despondent and help restore their confidence. As one young woman put it, ‘I had friends who stuck by me because they knew I was in a really bad way, really bad. And so they would say, Come on, let’s go and have some fun!’. (Young woman, GD).

Having fun, talking about a range of topics, going out with friends, chatting on the phone, letting oneself be loved, and being able to let off steam, are all ways the young people expressed what they do among themselves to cope with situations of adversity. For some young people, a wide circle of friends unquestionably makes it easier to manage emotional distress.

According to the young people, however, some differences in coping strategies are gender related. With some exceptions and personal individualities, they generally thought that young women were more expressive, more likely to talk over their problems with each other, and offered each other more mutual support. The externalization of the problem is linked to the idea that women are more sensitive, more vulnerable and more willing to show their feelings, and are more likely to express themselves by crying in situations of adversity.

In contrast, young men were thought to be stronger and more inclined to isolation or keeping the problem to themselves. They are more likely to externalize by acting on the defensive, sometimes violently, or increasing their consumption of alcohol, cigarettes or drugs.

These perceptions, expressed by the young people, are summarized in the words of one of the informants:

A boy won’t tell you the first time you ask him; he won’t spend all day down in the dumps or crying or whatever. But girls will. If something goes bad for them they’ll end up crying. I don’t know. They are weaker, if you will. Well, it depends on the person too. If a girl doesn’t want to tell you, then she won’t. With a boy, if you dig a bit, then he’ll tell you. (Young woman, GC).

Even when we take into account these differences, the participants said that closeness between peers fosters the trust that is necessary to express what one is feeling and experiencing. A friend ‘puts him or herself in your shoes’ better than a psychologist or another professional. Certainly, as the same young woman said, ‘Your friends are the best thing in this world’. (Young woman, GC).

### The affective and formative environment

As well as personal autonomy and strengthening sociability with peers, the young people also considered the support of the adult world as one of the resources to cope with distress. In this context parents, followed by teachers, are particularly significant.

Overall, the young people identified their parents as the main source of instrumental support in crisis situations. The young people turn to their parents when they face an economic difficulty, in the case of illness, or when they have to make important personal decisions concerning their education, work, the purchase of property, and so forth. As they stated in their narratives: ‘If you have a good level of trust with your parents, then your parents first’. (Young woman, SPD).

Communication between parents and children in the end is critical, it is essential, even though it’s hard, because of age, because of the figure they represent, because of the things you can’t tell them, but if we were all a bit smarter and talked about things more, everything would be better, we’d see that, that we are not alone … if something were to hurt me … my parents would be the right people. (Young man, GC).

The young people considered their parents as a suitable resource that allowed them to deal with situations of adversity because of what their parents know about them and because they can help from the experience that comes with age. The aspects the young people valued most positively about their parents’ help were associated with their presence and the fact that they can make things easier. They want their parents to stand by them in these situations, but without being over controlling and stifling. What they want most from their parents in these moments is love and affection. They viewed their parent’s unconditional love as the stimulus they need to cope with adversity. In the words of one informant:

When you are really down and your mum phones, and she calls and says, hey Ana what’s the matter, and you go and see her and she gives you a hug, or she’s on the couch and you lie in her lap and she strokes you. Or you go to see your dad, and you lie down next to him and fall asleep. It’s the little things that make you say it’s worth the effort to go on. (Young woman, GD).

With the exception of some gender differences, young people tend to choose their mothers to share their concerns and experiences with. Their narratives showed that they place more trust in their mothers and that they show more empathy. Some of them highlighted the unconditionally shown by their mothers. One young woman said, ‘Whatever I did she was always by me. Even when I did something wrong, she told me off or something, but she was still there’. (Young woman, SPD).

The young people viewed this idea of presence, reflected in the last words of the previous informant, as a necessary condition in their parents, although they also expressed the need for understanding from them. Above all they want to be understood and listened to. They want involvement in the relationship and the parent to be someone to trust rather than an authority to be avoided.

It is noteworthy that some of the young people with a diagnosis of depression recognized their parents as ‘expert professionals’ and ‘the best psychologists’. (Young woman, GD). ‘And my parents, well … a father and a mother can always give you the best advice’. (Young woman, GD). ‘My father is a great support, really, because he’s really good at talking to me, he always explains things and I understand him pretty well. He’s like a psychologist, if you like’. (Young man, GD).

In the educational context, the young people also viewed teachers as a resource to turn to, especially when the source of their distress was related to their studies and education. The young men in the diagnosed group are the exception, however; none of them referred to teachers as a potential self-care resource. This might suggest that the source of their distress does not lie in the context of education and for this reason they do not turn to teachers. For the others, ‘if it’s a problem with your studies, try and improve, talk to the right teachers so they can explain how you can get back on the right track’. (Young woman, GC).

In summary, we find that the presence of people close to the young people’s adult world, their unconditional love, the knowledge that comes with age, and trust establish them as a support resource for the young people of Catalonia.

### Externalization of the problem

Externalizing the problem involves the expansion of young people’s horizons to include self-care resources and actions that, to some extent, free them from the stress of the emotional distress.

As well as friends, parents or other adults, the young people might ask for help from other family members including siblings, grandparents, cousins, uncles and aunts. When the issue is ‘very personal’, talking to a brother or sister is an optimal choice; if the question concerns legal or administrative procedures, they might turn to an uncle or aunt, a cousin or other relative with expert knowledge in the area.

Whatever the case, when they identified the truly important people in their lives, after parents, the young people mentioned other family members, emphasizing these links over friendship links. In the words of some of the informants: ‘A friend can always let you down, but with the family you always know they are there for anything’. (Young man, GD).

I used to sideline my family a bit. And that’s the last thing you should do! Before, I preferred to go around with my so-called friends than stay at home and spend a bit of time with my sister, with my parents … Then I realized that no … that wasn’t a good thing. (Young woman, GC).

These comments show that the family is considered as a source of lasting relationships, despite possible conflicts or lack of understanding that can arise between family members. As one of the young men in our study felt, although parents sometimes do not listen or do not want to listen to their children as much as they would like, there is always a brother, sister, or cousin to turn to. In short, ‘you ask for help … first of all from a relative … Before you go to a friend or whoever, if it’s something critical … talk with the family. Because they know you best’. (Young man, SPD).

They understand that sharing the situation with someone close is a way of ‘getting the problem out’ and it is a way to hear other opinions to compare with their own. Externalization is based above all on the chance to talk to someone close and trusted and express through language emotions, feelings, concerns, wishes, and so forth.

The language young people use to externalize is not only spoken language, however; the language of the body also plays its part. Many young people refer to exercise or physical activity as a self-care strategy to channel the stress that the situation of adversity can cause.

Through exercise, the young people manage to ‘forget about the problem’, escape from it or be distracted for a while. They stated that sport was not only a way to be physically active, but also mentally active in that it helps them to ‘switch off from your problems’, the body becomes physical tired and ‘it is a way of disconnecting’.

The most popular sport among the young people in the study is basketball; young men and young women in all three groups referred to this team sport. A notable aspect of this sport, or any other team sport, is related to competitiveness. In the words of one informant:

As people, we need a shot of competitiveness and competition is good. Being able to compete with other people is good, because sometimes you win and sometimes you lose and if you lose you also learn how to cope with it. It’s like life, sometimes you win and you have to know how to win and other times you lose and you have to know how to react. (Young man, SPD).

From this young man’s account we can draw parallels between some aspects that the situations of adversity generate in the young people’s daily lives, and resolving conflicts when, as in this case, they play a sport.

For some young people, team sports were also a way to expand social networks and find support outside friendship and family circles. In this regard young people expressed their need to belong through the group dimension that sports offer. For some, sport also boosted their self-esteem. One of the young men said:

[Basketball] gives you values for yourself or … or it’s like, you feel too … like people care about you, right? Like … you are someone. You’re part of the group, you’re part of something and you’re important to other people … it’s what I said before: you feel loved! (Young man, GD).

### Sensory and aesthetic expression

Other self-care strategies the young people used were related to a whole range of activities that connect their internal and external worlds, and are essentially creative, artistic and sensory.

Some of the young people are members of a band, sing or play an instrument, or participate in drama or dance activities. They considered these activities to be much more than entertainment; they enabled them see reality in a different way, lift their mood and alleviate their distress. ‘I’m in a band because sometimes, with some songs, I can really let go, more than anything, I let off steam and it’s a … because I’m distracted. So I don’t think about … I don’t know, maybe it’s like a therapy’. (Young man, GD).

Music is the most important thing in the whole world for me. Because music always knows how you are and sometimes tells you how you feel. It can do anything because there are lots of kinds of music, of songs, and you’ll always find something that helps you. Because you hear a song and after that you feel better. The messages you get normally match the music. The music I listen to has a lot of guitar solos and sometimes just one guitar solo can give you some amazing feelings. (Young man, SPD).

[Theater] gives me a new way of seeing things, because it puts you in another person’s shoes, when you’re performing … and … you end up seeing things in a different way, you know? One day you see things more from a child’s perspective, another day, from the perspective more as if you were a more stingy person, for instance, you know? And … and … I understand people a lot more … when you see that the person on stage isn’t you, that it’s a character … it’s like it’s much better! (Young woman, GD).

The experience of living another reality opens up the idea of living in the present, the ‘here and now’. In this line, some of the young people mentioned they would recommend, or have already recommended, that their friends try relaxation and meditation techniques, art therapy or laughter therapy when they are in a situation of adversity. In general, without evaluating these strategies negatively, they stated that the benefit they derive from them is momentary.

I don’t think it gets to the root of the problem at all. It’s just to make you feel better. I would do it as an alternative therapy. Yeah, I’m not sure, but something like that. I think it can do you a lot of good for an afternoon, but maybe the next morning you’re depressed again because the problems are still there. (Young woman, GD).

They took a similar line on the effects of other techniques or practices aimed to stimulate the body’s own healing abilities, such as naturopathy, homeopathy or reflexology. Some participants from the clinical diagnosis or self-perceived distress groups occasionally used these techniques.

I did reflexology, and well, at the time it helped me a lot, because I am very nervous, and it was relaxing and whatever, but it’s the same as the psychologist, they can help you, but they won’t solve your problems. (Young woman, SPD).

I was under a lot of pressure, and I had all the stress of my studies, so I went there to color therapy and Bach flower remedies, and it was really good … I don’t know, but for me it was. Sure, it depends on each person, it works for some people but not for others, and some people don’t want to believe in it and it works for others because they believe in it … you know? It’s like it’s all relative, but I think it’s a good alternative, you’re there and it’s you who reflects, it’s not someone talking to you or trying to get stuff out of you, or … I don’t know. I think … like I suppose because of cultural views it’s different, because going to the psychologist, it’s like there’s something wrong with you! But you go to the homeopath or something and a lot of people go there, just for their wellbeing, because … I don’t know … you’ve had a bad week or whatever … and it’s different. (Young woman, GD).

Despite the sporadic or transient benefits that these health practices might offer, the young people who use them preferred this option to others from allopathic medicine.

### Self-care strategies and subgroups

**Figure 2:**
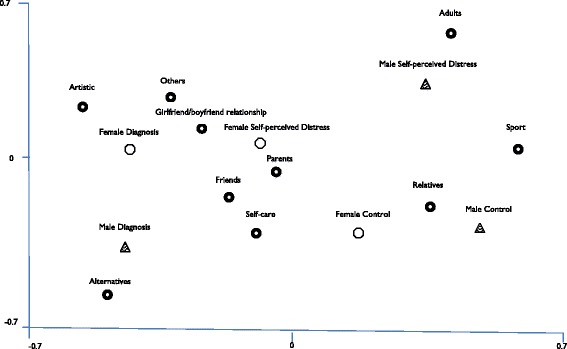
**Strategies of self-care and subgroups.** Note: Analysis using data from Table 2. Prepared using UCINET 6.454, model: coordinates.

A partial association between the four categories designed with the self-care strategies the young people identified, and the three subgroups (GD, SPD and GC) emerged from a correspondence analysis. Figure 2 shows the following distribution:

1. The young men in the diagnosis subgroup are associated with the alternatives item.
2. The young women in the diagnosis subgroup are associated with artistic and other items.
3. Young women with self-perceived distress are associated with the resources in the ‘between individuality and sociability’ category (own self-care, friends and others), and adults (parents and partner).
4. Young men with self-perceived distress are closer to adults.
5. The young women in the control group are closer to relatives.
6. The young men in the control group are closer to the sports strategy.

All the self-care strategies described were cited, in general, by all the young people. The above distribution of the sample subgroups in relation to self-care resources, however, reveals certain trends indicating the therapeutic pathways that young people take. The four sets of self-care resources discussed above can be interpreted on a scale in which the resources related to individuality and sociability form the first level of the pathway, and sensory and artistic expression is related to the resources mobilized in the last level, in other words, when the previous resources have also been used.

In line with this idea, the young people’s pathways allowed us to identify the use they make of the various self-care resources. The trend revealed is that young men and women with a diagnosis follow the entire sequence and pass through all the identified levels, to eventually use resources related to sensory and artistic expression. In fact, the young people in the group with a diagnosis were more likely to use resources or alternative therapies, and the young women in this group tended toward activities associated with art. We find a predisposition among young people with a diagnosis to look outside their social network (parents, friends, family) for self-care strategies.

For their part, the self-perceived distress group tends to focus more on the resources identified between individuality and sociability, and those relating to the adult world. In contrast to the group with a diagnosis, the self-perceived distress group mainly activated their social networks as a self-care resource. Mobilizing the level of resources related to externalizing the problem is linked to the pathway followed by the young people in the control group.

## Discussion

In this study we show, from a qualitative perspective, the various self-care resources young people use in Catalonia (Spain) to cope with events of depressive distress and anxiety. Our analysis revealed ten main resources, which we grouped into four areas covering different levels and potential therapeutic pathways young people take, depending on whether they have a formal diagnosis, self-perceive distress or, the case of the control group, neither of the above.

The young people’s narratives showed that they experience distress as their own responsibility. For this reason they felt able to resolve their distress through their own resources. As we have shown, their strategies ranged from their individuality to sociability expressed through their relationships with others (friends, parents, families, adults, etc.), membership of groups with varying levels of organization (bands, drama groups, sports teams, etc.), or other self-care strategies identified at a more sensory (relaxation, meditation, laughter therapy) or alternative level (naturopathy, homeopathy, reflexology). The young people’s perception of self-sufficiency can be considered as reinforcing the use of this variety of lay resources.

Even when this range of strategies is taken into account, we note that young Catalans prioritized seeking help from friends and family to cope with their distress. This finding coincides with other studies [14]. Indeed, our research confirms that talking to a close friend or family member, and maintaining contact with these social networks, are actions young people considered to prevent or apply in situations of depressive distress or similar states [13,28,29]. The study also highlights the ambivalence, however, between the sense of self-care reflected in the level of discourse, and the relative dependence, in the real dimension, on their social and family networks. In fact, some scholars have considered young people’s conviction that they can resolve their mental health problems on their own to be a barrier to seeking professional help [13,30,31]. Other studies have also suggested that young people use these support networks with such frequency because they are readily accessible and easily put to effect. This resource is, in the end, economic and involves less stigmatization of the problem [12]. Whatever the case, young people see social and family networks as another link in the chain of resources in their immediate world [22,24]. Indeed, young people use these relationships to build ideal pathways of attention and care consistent with their representations of personal autonomy and lay problem solving strategies [32].

In this context, we believe that the young Catalans’ choice of lay care strategies should be understood as a social reality that informs us how this population group copes with their situations of distress, while at the same time revealing key ways to address the young people’s mental health by placing them at the center of and making them active parties in their own processes.

The frequency with which the young people’s narratives refer to terms such as presence, understanding, closeness and trust to define the relationship they have with or expect from the other (friend, parent, relative or teacher) reveals these to be desirable qualities in the listener and the person with whom they share their distress. It is also a sign that there is a real need for the young people to establish a dialogic and intersubjective encounter with the other to restore their well-being and strengthen their independence. For young people this relationship is based on the understanding and peace of mind to narrate, express and feel their pain.

What they expect from their interlocutors in an ideal situation appears to take the form of *being there*, of letting them talk, listening and giving emotional support without damaging their self-esteem and autonomy. We understand that for the young people this *being there* means that their local-subjective world is valued, and that they are accompanied in a process in which the subject him or herself predominates, and is not seen as afflicted with distress or reflecting a diagnostic category. Paying attention to the diagnosis above all else unquestionably induces the risk of prioritizing prompt treatment; treatment that is not necessarily effective just because it is fast, and cannot always recognize the capacity for self-management our study population has.

## Conclusions

Our findings shed light on young people’s experience in using lay resources for depressive distress or similar situations. In many cases the lay strategies our study population identified mitigate and resolve distress, although not necessarily to the exclusion of professional care actions. The willingness to use social networks, play in a band, play sport, write or meditate, among others, shows the capacity and ability young people have to address their mental health problems, thereby enhancing their sense of self-sufficiency. Taking these aspects into account highlights the need to be sensitive in considering the lay strategies young people use as an additional option in mental health care. Their active efforts to retain control and manage their abilities to resolve the distress process should also be taken into account.

From this perspective, we consider that professionals and the health policies and strategies implemented in Catalonia should respect, consider and be constructed on the basis of young people’s sense of self-sufficiency. The self-care centered on the young person’s sociability is also an important factor, and professionals should take into account social relationships with friends, parents, family, and so forth when determining what help is needed and establishing educational programs. As the study has highlighted, young people expect and want to be heard, encouraged and accompanied. In their way, they are calling for the closeness and presence of the other. Professionals should therefore first recognize these key issues and apply them when they attend to young people.

## Additional file

Additional file 1:
**The vignette and qualitative interview.** Additional file 1 details the script of the questions used in the semi-structured interview following the reading of the vignette which was designed specifically for the research.
