# Supplementary material for: Self-care strategies for emotional distress among young adults in Catalonia: a qualitative study
Source: Int J Ment Health Syst. 2015 Mar 10;9:9. doi: 10.1186/s13033-015-0001-2 (PMC4363191; doi:10.1186/s13033-015-0001-2)
Supplement: Additional file 1: — The vignette and qualitative interview. Additional file 1 details the script of the questions used in the semi-structured interview following the reading of the vignette which was designed specifically for the research. [file 13033_2015_1_MOESM1_ESM.pdf]

Appendix 1: The vignette and qualitative interview

Mary/John is 17 and has felt bad for the last two weeks. She/he feels sad and empty and sometimes irritable. She/he finds it very difficult to concentrate. She/he no longer finds pleasure in the things that she/he has always enjoyed and has lost her/his appetite. She/he finds it very difficult to sleep at night and has no energy during the day to carry out his/her normal activities.

1. What do you think is wrong with Mary/John?
2. Which different names are given to what is happening to her/him?
3. Can you tell me which words people your age use to talk about these states?
4. Have you ever found yourself in this situation or in a similar one? If you have, can you describe it to us? If not, do you know anyone your age who has been in this situation? If so, how do you remember that situation? How old were you?
5. What could be the causes of Mary/John's condition?
6. Do you think girls and boys experience emotional distress for different reasons?
7. If you have ever felt this way, what caused it?
8. Do you think that emotional distress is experienced differently when you are 15 than when you are 18? Why?
9. How can you recognize when somebody, either a boy or a girl, is in the same situation as Mary/John (gestures, facial expression, attitude, etc.)?

10. Do you think the signs of emotional distress are different in boys and girls? How?
11. How do you know when you are feeling bad? What happens to you? How do you feel? What do you do? What do you stop doing? How well does this work for you?
12. What could Mary/John do to feel better? (list the possibilities)
13. How could she/he be helped? (list the possibilities)
14. If the respondent does not mention any health professional, say to him/her: Mary/John does not want to go to a health professional for help. Why do you think this is?
15. What professional health resources/people do you know of? (list them)
16. What do you think about each of these options?
17. Which ones would you recommend to Mary/John? Why?
18. Have any of your friends ever been to see any of these professionals? (If the answer is yes) How do you know? Did they tell you? How did it go for them? (well, badly, etc.); (If the answer is no) Why do you think they haven't gone?
19. Would you go to one of these professionals if you felt like Mary/John? Why? What would you expect from these professionals?
20. If you decided not to go to one of these professionals, who would you go to for help (person, group/institution, etc.)? Why?
21. If you went to one of these professionals, would you go to more than one person/group/institution, etc.? Why?

22. Has any member of your family ever been to see one of these professionals? (if the answer is yes) How did it go for them? (if the answer is no) Why haven't they gone?
23. What does your family think about these professional resources?
24. What would you do if you were Mary's/John's friend?
25. What could her/his family do to help her/him?
26. Which strategies do you think you use most often to deal with your problems?
27. Which strategies do your friends use?
28. Which strategies does your family use?
29. What would you like others to do to help you feel better?
30. Do you belong to any youth association, choral society, hiking group, church-affiliated group, dance group, art club, or other voluntary association?
31. If so, which one(s)? Why? What do you get out of it? How do you feel in these groups?
32. If not, why? Have you ever belonged to any group of this kind?
33. If so, which one(s)? When? For how long? Why did you join? How did you feel in the group? When did you leave? Why did you leave?
34. Do you have the same number of friends now as you had when you were 15? If the number of friends you have has changed, why do you think this is?
35. Do you think this has changed the way you think about friendship?
36. Do you tell your friends more things now than you used to? Why do you think this is?

37. Have the kinds of things you talk about with your friends changed? If so, why do you think this is?
38. Have the things that worry you changed since you were 15? If so, what did they used to be? What are they now? Why do you think this change has happened?
